# Sigmoid volvulus: An update for Atamanalp classification

**DOI:** 10.12669/pjms.36.5.2320

**Published:** 2020

**Authors:** Sabri Selcuk Atamanalp

**Affiliations:** 1 Prof. Sabri Selcuk Atamanalp, MD. Department of General Surgery, Faculty of Medicine, Ataturk University, 25040, Erzurum, Turkey

**Keywords:** Classification, Prognosis, Sigmoid colon, Treatment, Volvulus

## Abstract

Sigmoid volvulus (SV), the wrapping of the sigmoid colon around itself, is a rare colonic obstruction form. SV requires emergency endoscopic or surgical treatment following an urgent resuscitation and the prognosis is relatively poor. Although many different classifications have been made for SV in the past, the unique classification system, which assists the decision making in the management in addition to the estimation of the prognosis, was described in 2017. Thereafter, a great number of relevant subjects were discussed in the literature. The aim of this study is to update the present classification system in light of above-mentioned discussions and our experience with 1,028 SV patients, which is the largest single-centre SV series over the world.

## INTRODUCTION

Sigmoid volvulus (SV) is the wrapping of the sigmoid colon around itself causing a colonic obstruction. Following the description of SV by Rokitansky in 1836, many different classifications including clinical course (acute, subacute or chronic), severity (complete or incomplete), prevalence (sporadic or endemic), torsion direction (clockwise or counterclockwise), and torsion degree (<360, 360, or >360) have been made in literature.^1^ However, none of these classifications provides practical information about the treatment or prognosis of SV. Finally, in 2017, a new classification, treatment algorithm, and prognosis-estimating system were described for SV. In this classification system, age, American Society of Anesthesiologists (ASA) score, and the local condition of bowels were used as parameters.^2^ Thereafter, a great number of relevant subjects were evaluated by different authors.^3^ Although SV is rare worldwide, it is endemic in my practice area, Eastern Turkey. I and my colleagues have a 1,028-case experience with SV over 53.5 years (June 1966 to January 2020), which is the largest single-center SV series in the world.^3^ I wanted to utilize our comprehensive experience to evaluate and upgrade the classification of SV.

## CLINICAL EXPERIENCE

I reviewed the clinical records of 1,028 SV patients retrospectively until June 1986 and prospectively thereafter. This study was approved by the Institutional Ethics Committee (Ataturk University, Faculty of Medicine, February 23, 2017, B.30.2.ATA. 0.01.00/16). In our series, non-operative treatment was used in 750 patients (77.9%) with 82.7% success, 0.7% mortality, 2.3% morbidity, and 5.5% early recurrence rates. Of patients, 476 (46.3%) were treated by emergency surgery with 16.8% mortality, 34.2% morbidity, and 0.6% early recurrence rates. Elective surgical treatment was used in 114 of endoscopically decompressed 584 patients (19.5%) with 0.0% mortality, 11.4% morbidity, and 0.0% early recurrence rates.

## DISCUSSION

SV tends to recur for life in addition to its grave prognosis.^4^ It is clear that uncomplicated and non-gangrenous SV patients are treated by endoscopic decompression, while emergency surgery is required in complicated and gangrenous patients, or in whom endoscopic decompression is unsuccessful. In a limited number of patients, emergency or elective percutaneous endoscopic colopexy (PEC) may be preferred. Additionally, elective surgery is suggested in some selected endoscopically decompressed patients.^5^

Firstly, the ASA score is an important factor in the decision-making process.^2^ In my experience, elective surgery may be suggested in patients with ASA I-III scores, in whom operative mortality is lower than that of conservation. Secondly, age is another important factor.^6^ Nevertheless, life expectancy varies from a country to another and it is generally higher in developed countries. As known, SV is more common in undeveloped or developing countries.^1^ Additionally, average life decreases in time. For this reason, I prefer ‘life expectancy’ as a checkpoint instead of ‘specified age limit’. For example, in Turkey, life expectancy is 75 years for men, and I may accept the age limit as 75. Thirdly, when evaluated in the beginning stages of the operation, the local bowel condition, including borderline ischemia, edema, and difference in proximal and distal bowel diameters arising from extremely dilatation, may misguide.^7^ Therefore, if needed, I suggest the ex-post evaluation after the resection to obtain a proper action. Finally, following a successful endoscopic detorsion, PEC is suggested in some selected patients.^8^ I propose PEC a day or two later, as an elective process, in selected patients with ASA IV-V scores. This period may allow an improvement in the general condition of inoperable cases. The results of this updated classification, which may assist the decision making in the management in addition to the estimation of the prognosis is summarized in Table-I.

**Table-I:** Atamanalp classification for sigmoid volvulus.

Group	Definition	Treatment	Mortality (%)	Morbidity (%)	Recurrence (%)
I A	G 0, A 0, ASA I-III	Endoscopic decompression	0-1	1-2	15-55
		or plus elective surgical resection and anastomosis	0-2	5-15	0-1
I B	G 0, A I or ASA IV-V	Endoscopic decompression	5-10	10-25	15-55
		or plus percutaneous endoscopic colopexy	8-15	15-30	0-15
		or plus elective percutaneous endoscopic colopexy	5-13	13-28	0-15
II A	G 0, A 0, ASA I-III, E I	Surgical decompression	1-5	5-15	15-55
		or plus surgical colopexy or mesopexy or mesoplasty	1-8	10-20	10-20
		or plus surgical resection and anastomosis	1-10	15-25	0-1
II B	G 0, A I or ASA IV-V, E I	Surgical decompression	10-30	20-40	15-55
III A	G I, A 0, ASA I-III, B 0	Surgical resection and anastomosis	5-10	10-30	0-1
III B	G I, A I or ASA IV-V or BI	Surgical resection and stoma	20-30	30-60	0-1

A 0, age < life expectancy; A I, age ≥ life expectancy; ASA I, patient with no other disease; ASA II, patient with mild systemic disease;

ASA III, patient with severe systemic disease; ASA IV, patient with life-threating systemic disease; ASA V, moribund patient;

B 0, normal bowel; B I perforated bowel or borderline ischemic, edematous or differential scaled bowel ends following resection;

E I, unsuccessful endoscopy; G 0, viable bowel, G I, gangrenous bowel.

## CONCLUSION

Following the diagnosis and resuscitation, by using the upgraded classification system, patients with SV may be distributed into groups in 30 seconds. In the next 15 seconds, the management option may be decided and a conclusion about the prognosis may be reached in the subsequent 15 seconds. In this way, the theoretical evaluation may be solved in approximately a minute and practical management may be performed without loss of time.
